# Furosemide exacerbated the impairment of renal function, oxygenation and medullary damage in a rat model of renal ischemia/reperfusion induced AKI

**DOI:** 10.1186/s40635-023-00509-3

**Published:** 2023-05-01

**Authors:** Olcay Dilken, Can Ince, Aysegul Kapucu, Paul M. Heeman, Bülent Ergin

**Affiliations:** 1 Laboratory of Translational Intensive Care, Department of Intensive Care Adult, Erasmus MC, University Medical Center Rotterdam, Erasmus University, Doctor Molewaterplein 40, 3015 GD Rotterdam, The Netherlands; 2 Department of Intensive Care, Faculty of Medicine, University of Istanbul-Cerrahpasa, Istanbul, Turkey; 3 Department of Zoology, Faculty of Science, University of Istanbul, Istanbul, Turkey; 4 Department of Medical Technical Innovation & Development (MIO), Amsterdam University Medical Centre (UMC) Location AMC, University of Amsterdam, Amsterdam, The Netherlands

**Keywords:** Acute kidney injury, Renal failure, Furosemide, Urine output, Creatinine, Oxygenation

## Abstract

**Background:**

Perioperative acute kidney injury (AKI) caused by ischemia–reperfusion (IR) is a significant contributor to mortality and morbidity after major surgery. Furosemide is commonly used in postoperative patients to promote diuresis and reduce tissue edema. However, the effects of furosemide on renal microcirculation, oxygenation and function are poorly understood during perioperative period following ischemic insult. Herein, we investigated the effects of furosemide in rats subjected IR insult.

**Methods:**

24 Wistar albino rats were divided into 4 groups, with 6 in each; Sham-operated Control (C), Control + Furosemide (C + F), ischemia/reperfusion (IR), and IR + F. After induction of anesthesia (BL), supra-aortic occlusion was applied to IR and IR + F groups for 45 min followed by ongoing reperfusion for 15 min (T1) and 2 h (T2). Furosemide infusion was initiated simultaneously in the intervention groups after ischemia. Renal blood flow (RBF), vascular resistance (RVR), oxygen delivery (DO_2ren_) and consumption (VO_2ren_), sodium reabsorption (TNa^+^), oxygen utilization efficiency (VO_2_/TNa^+^), cortical (CμO_2_) and medullary (MμO_2_) microvascular oxygen pressures, urine output (UO) and creatinine clearance (Ccr) were measured. Biomarkers of inflammation, oxidative and nitrosative stress were measured and kidneys were harvested for histological analysis.

**Results:**

IR significantly decreased RBF, mainly by increasing RVR, which was exacerbated in the IR + F group at T2 (2198 ± 879 vs 4233 ± 2636 dyne/s/cm^5^, p = 0.07). CμO_2_ (61.6 ± 6.8 vs 86 ± 6.6 mmHg) and MμO_2 _(51.1 ± 4.1 vs 68.7 ± 4.9 mmHg, p < 0.05) were both reduced after IR and did not improve by furosemide. Moreover, VO_2_/TNa^+ ^increased in the IR + F group at T2 with respect to the IR group (IR: 3.3 ± 2 vs IR + F: 8.2 ± 10 p = 0.07) suggesting a possible deterioration of oxygen utilization. Ccr did not change, but plasma creatinine increased significantly in IR + F groups. Histopathology revealed widespread damage both in the cortex and medulla in IR, IR + F and C + F groups.

**Conclusion:**

Renal microvascular oxygenation, renal function, renal vascular resistance, oxygen utilization and damage were not improved by furosemide administration after IR insult. Our study suggests that furosemide may cause additional structural and functional impairment to the kidney following ischemic injury and should be used with caution.

## Background

Renal damage and loss of function can frequently occur during and after abdominal and cardiovascular surgery, transplantation, and renal arterial reconstruction surgery due to ischemia/reperfusion (IR) induced acute kidney injury (AKI) [1–4].

The kidney is vulnerable both to ischemic and hypoxic injury, due to its high energy expenditure and workload. The renal blood flow is continuously adjusted with strict autoregulatory mechanisms in physiological states to ensure a balance between oxygen delivery and consumption [5]. However, this balance can be impaired after IR injury. Renal tubular damage is triggered by the imbalance between oxygen delivery and consumption, hypoxia, inflammation, and it is further exacerbated by hyperoxia and oxidative stress during reperfusion [6].

Furosemide is a diuretic drug which inhibits the electrolyte transport receptors in the thick ascending limb (TAL) of the Henle. Furosemide can increase the excretion of Na^+^ and water by inhibiting Na–K–2Cl co-transporter in TAL, therefore it reduces Na–K-ATPase-mediated oxygen consumption. It is well known that fluid overload in patients with AKI is independently associated with mortality and poor outcome [7, 8]. Therefore, furosemide is commonly used to promote diuresis in the postoperative and critically ill patients with fluid overload, regardless of whether the patient has AKI or not. However, human and animal studies have shown that furosemide is not effective in preventing AKI in these settings [9, 10]. Furthermore, it was recently demonstrated that diuretic use was independently associated with higher AKI risk in patients hospitalized with Covid-19, which is a hyperinflammatory disease per se [11]. Several mechanisms including increased oxidative stress, relative hypovolemia, and higher renal vascular resistance are suggested as factors causing AKI following furosemide use [12, 13].

We hypothesized that the use of furosemide in perioperative phase may have some beneficial effect to reduce renal damage triggered by oxidative stress and hypoxia because its mechanism of action to reduce the medullary oxygen consumption as a result of increasing Na^+^ and water excretion. However, the existing research fails to resolve the impact of furosemide on IR-induced AKI in perioperative period. Here, we believed that understanding the effects of furosemide on the kidney, which is already damaged, may provide a new insight to use of furosemide in postoperative and ICU patients with renal injury. Therefore, we aimed to investigate the effects of furosemide on the renal cortical and medullary microvascular oxygenation, function, inflammation and extent of histopathological damage following a model of supra-aortic occlusion induced AKI in rats.

## Methods

The current study uses an experimental design to simulate IR-induced AKI after cardiovascular or abdominal surgery. Oxygen phosphorescence technique employed in this study offers the advantage of the measurement of individual oxygen concentration both in the renal cortex and the medulla.

### Animals

All experiments described in this study were approved by the Institutional Animal Experimentation Committee of the Academic Medical Centre of the University of Amsterdam (DFL83). Care and handling of the animals were following the guidelines for Institutional and Animal Care and Use Committees. The study has been carried out according to the Declaration of Helsinki. Experiments were performed on 24 male Wistar albino rats (Charles River, The Netherlands) with a mean ± SD bodyweight of 340 ± 26 g.

### Surgical preparation

All the animals were anesthetized with an intraperitoneal injection of a mixture of 90 mg/kg ketamine (Nimatek, Eurovet, Bladel, The Netherlands), 0.5 mg/kg dexmedetomidine (Dexdomitor, Pfizer Animal Health BV, Capelle aan den IJssel, The Netherlands), and 0.05 mg/kg atropine-sulfate (Centrafarm Pharmaceuticals BV, Etten-Leur, The Netherlands). After preparing a tracheotomy, the animals were mechanically ventilated with a mixture of air and oxygen with a FiO_2_ of 0.4. Body temperature was maintained at 37 ± 0.5 °C during the entire experiment by an external thermal heating pad. Ventilator settings were adjusted to maintain end-tidal pCO_2_ between 30 and 35 mm Hg and arterial pCO_2_ between 35 mm Hg and 40 mm Hg.

For drug and fluid administration and hemodynamic monitoring, vessels were cannulated with polyethylene catheters with an outer diameter of 0.9 mm (Braun, Melsungen, Germany). A catheter in the right carotid artery was connected to a pressure transducer to monitor mean arterial blood pressure (MAP). The right jugular vein was cannulated for continuous infusion of Ringer’s Lactate (Baxter, Utrecht, The Netherlands) at a rate of 15 ml/kg/h to continuous fluid resuscitation and 50 mg/kg/h ketamine dissolved in 5 ml Ringer’s Lactate for the maintenance of anesthesia. The right femoral artery was cannulated for withdrawing blood samples and the right femoral vein for drug administration.

The left kidney was exposed, decapsulated, and immobilized in a Lucite kidney cup (K. Effenbergerite, Pfaffingen, Germany) via a 4-cm left flank incision of each animal. Renal vessels were carefully separated from surrounding tissue, nerves, and adrenal gland. A perivascular ultrasonic transient time flow probe was placed around the left renal artery (type 0.7 RB Transonic Systems Inc, Ithaca, NY) (T206, Transonic Systems Inc, Ithaca, NY) to simultaneously measure RBF. The left ureter was isolated, ligated, and cannulated with a polyethylene catheter for urine collection.

After surgical preparation, an optical fiber was placed 1 mm above the decapsulated kidney and another optical fiber was placed 1 mm on the renal vein to measure renal microvascular and venous oxygen pressures using phosphorimetry, respectively. Oxyphor G2, a two-layer glutamate dendrimer of tetra-(4-carboxy-phenyl) benzoporphyrin (Oxygen Enterprises Ltd, Philadelphia, PA) was subsequently infused (6 mg/kg IV over 5 min) followed by 30 min of stabilization time. The surgical field was covered with a humidified gauze compress throughout the entire experiment to prevent drying of the exposed tissues.

### Experimental protocol

The rats were randomly divided into four groups (n = 6 per group determined by the power analysis (nQuery advisor, GraphPad Software DBA Statistical Solutions, San Diego): a sham-operated time control group (C), a group subjected to renal ischemia for 45 min by supra-aortic occlusion with a custom-made vascular occluder placed in between the left renal artery and superior mesenteric artery followed by 2 h of reperfusion (IR), a group subjected to renal ischemia for 45 min and followed by 2 h of reperfusion in which furosemide 50 µγ/kg /h was continuously administered after the release of the clamp (IR + F), and finally a sham-operated group in which furosemide was administered to the control group (C + F). At the end of the experiments, renal tissue samples were harvested and stored in both %4 formaldehyde solution for histological analysis and – 80 °C for a marker of oxidative stress, inflammation, and nitric oxide levels.

### Blood and plasma variables

Arterial blood samples (0.25 ml) were drawn from the femoral artery at three-time points: before aortic occlusion (baseline, BL); 15 min after reperfusion (initial reperfusion phase, T_1_); and 120 min after reperfusion (late reperfusion phase T_2_). The blood samples were replaced by the same volume of HES 130/0.4 (Voluven, 6% HES 130/0.4; Fresenius Kabi Schelle, Belgium). The samples were used for the determination of blood gas values, as well as for the determination of the hemoglobin concentration, hemoglobin oxygen saturation, and electrolytes concentrations (ABL80 Flex Blood Gas Analyzer, Radiometer, Copenhagen, Denmark). The plasma and urine samples were used for the measurement of creatinine levels.

### Renal microvascular and venous oxygenation

Microvascular oxygen tension in the renal cortex (CµPO_2_), outer medulla (MµPO_2_), and renal venous oxygen tension (P_rv_O_2_) were measured by oxygen-dependent quenching of phosphorescence lifetimes of the systematically infused albumin targeted (therefore circulation-confined) phosphorescent dye Oxyphor G2 [14]. Oxygen measurements based on phosphorescence lifetime techniques rely on the principle that phosphorescence can be quenched by energy transfer to oxygen resulting in the shortening of the phosphorescence lifetime. A linear relationship between reciprocal phosphorescence lifetime and oxygen tension (i.e., Stern–Volmer relation) allows quantitative measurement of PO_2_ [15].

### Calculation of derivative oxygenation parameters and renal vascular resistance

Arterial oxygen content (AOC) was calculated by the following equation: (1.31 × hemoglobin × SaO_2_) + (0.003 × PaO_2_), where SaO_2_ is arterial oxygen saturation and PaO_2_ is the arterial partial pressure of oxygen. Renal venous oxygen content (RVOC) was calculated as (1.31 × hemoglobin × SrvO_2_) + (0.003 × PrvO_2_), where SrvO_2_ is venous oxygen saturation and PrvO_2_ is renal vein partial pressure of oxygen (measured using phosphorimetry [14]. The SrvO_2ren_ was calculated using the Hill equation with P50 = 37 Torr (4.9 kPa) and Hill coefficient = 2.7. Renal oxygen delivery was calculated as DO_2ren_ (ml/min) = RBF x AOC. Renal oxygen consumption was calculated as VO_2ren_ (ml/min) = RBF (AOC-RVOC). An estimation of the renal vascular resistance (RVR) was made as: RVR (dynes/s/cm^5^) = (MAP/RBF) × 100.

### Assessment of kidney function

The high plasma creatinine level was accepted as a short-term AKI definition based on the KDIGO criteria. Creatinine clearance (Clear_crea_(ml/min)) was measured as an index of the glomerular filtration rate and calculated with the following formula: Clear_crea_ = (U_urea_ × V)/P_crea_, where U_crea_ was the concentration of creatinine in the urine, V is the urine volume per unit time, and P_crea_ was the concentration of creatinine in plasma. Additionally, excretion fraction of Na^+^ [EF_Na_ (%)] was calculated and used as a marker of tubular function in the following formula: EF_Na_ = (U_Na_ × P_crea_)/ (P_Na+_ × U_crea_) × 100, where U_Na_ was Na^+^ concentration in urine and P_Na_ was the Na^+^ concentration in plasma. The renal oxygen extraction ratio was calculated as O_2_ER_ren_ (%) = VO_2ren_/DO_2ren_ × 100. Clear_crea_ and EF_Na_ were determined at all time points. Furthermore, the renal energy efficiency for sodium transport (VO_2ren_/T_Na_) was assessed using a ratio portrayed by the total amount of VO_2ren_ over the total amount of sodium reabsorbed (T_Na_, mmol/min) that was calculated according to: (Clear_crea_ × P_Na_) – U_Na_ × V. This parameter can be regarded as an important variable reflecting tubular cell function and oxygen utilization.

### Measurement of inflammatory cytokines and glycocalyx component

Inflammatory cytokines TNF-α, IL-6, and hyaluronan (HA) (Rat TNF-a ELISA kit, DY510; Rat IL-6 ELISA kit, DY506 and Rat Hyaluronan Duoset ELISA kit, DY3614, R&D System Inc, Minneapolis, Minn) were determined by ELISA from renal frozen tissue samples. Malondialdehyde (MDA) was quantified using a Quattro Premier XE tandem mass spectrometer (MS/MS, Waters, Milford, Mass) with an Acquity sample manager and an Acquity binary solvent manager [16]. Nitric oxide levels were measured with chemiluminescence method by the Sievers NO analyzer [17]. The level of cytokines, MDA, NO and hyaluronan was expressed as per gram of protein (Bradford assay).

### Histological analysis

Kidney tissues were fixed in 4% formalin and embedded in paraffin. Kidney sections (4 µm) were deparaffinized with xylene and rehydrated with decreasing percentages of ethanol and finally with water. The kidney sections were stained with periodic acid–Schiff reagent (PAS) and hematoxylin–eosin. Histologic changes in the cortex and medulla were assessed by semi-quantitative grading score of tubular damage, necrosis and inflammation. The degree of tubular dilatation in medulla and cortex were estimated at 400× magnification using 10 randomly selected fields by the three-point scoring system: 1 indicates < 25% of tubules show dilatation; 2, dilatation covering 25% to 50% of tubules; 3, covering > 50% of tubules. Tubular necrosis was classified the four-point scoring system as 1 = less that 25% tubular necrosis, 2 = 25–50% necrosis, 3 = 50–75% necrosis and 4 = more than 75% tubular necrosis. Inflammatory score was analyzed by the area of infiltrating cell as 1, none; 2, less that 25% of area; 3, 25–50% of area; 4, 50–75% of area and 5, more than 75% of area (adapted from [18]).

### Statistical analysis

Data analysis and presentation were performed using GraphPad Prism 8 (GraphPad Software, San Diego, Calif). Shapiro–Wilk normality test was used for the Gaussian distribution of data. Values are reported as the mean ± SD. Two-way ANOVA for repeated measurements with a Tukey multiple comparison tests were used for comparative analysis of inter- and intra-group variations. The repeated-measures analysis of variance (one-way ANOVA with a Tukey multiple comparison test) was used for comparative analysis between the groups if baseline values differed distinctively. Statistical analysis of histological results (values are reported mean ± SE) was performed by one-way analysis of variance with Tukey multiple comparison test. *p*-value of < 0.05 was considered statistically significant.

## Results

### Systemic and renal hemodynamic variables

**Fig. 1:**
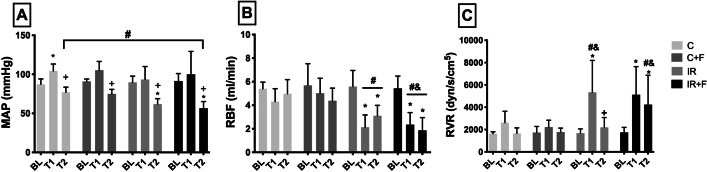
Alteration of mean arterial pressure (MAP) (**A**), renal blood flow (RBF) (**B**) and renal vascular resistance (RVR) (**C**) between the experimental groups and time points. Values are represented as mean ± SD, ^*^p < 0.05 vs. BL, ^+^*p* < 0.05 vs. T1, ^#^*p* < 0.05 vs. at same time point of Control (C) group, ^&^ < 0.05 vs. at same time point of Control + Furosemide (C + F) group

IR had a deleterious effect on all systemic and renal hemodynamic variables (Fig. 1). MAP and RBF were significantly reduced in the IR (MAP: 89.6 ± 8 mmHg at BL; 93.4 ± 16.6 mmHg at T1; 61.8 ± 6.9 mmHg at T2) (RBF: 5.5 ± 1.3 ml/min at BL; 2.1 ± 1 ml/min at T1 and 3.1 ± 0.9 ml/min at T2) and IR + F (MAP: 91.3 ± 9.6 mmHg at BL; 100 ± 29.3 mmHg at T1; 56.5 ± 8.6 mmHg at T2) (RBF: 5.4 ± 1 ml min at BL; 2.3 ± 1 ml/min at T1 and 1.8 ± 1 ml/min T2) groups at T2 in comparison to the BL and T1 (*p* < 0.05). Furosemide administration improved neither MAP nor RBF. RVR significantly increased in IR group at T1 (1678 ± 382 dyne/s/cm^5^ at BL vs 5338 ± 2860 dyne/s/cm^5^ at T1, *p* < 0.05) and IR + F group at T1 (1745 ± 452 dyne/s/cm^5^ at BL vs 5123 ± 2517 dyne/s/cm^5^ at T1, *p* < 0.05) and T2 (1745 ± 452 dyne/s/cm^5^ at BL vs 4223 ± 2636 dyne/s/cm^5^ at T2, *p* < 0.05) with respect to BL. RVR reduced in the IR group at T2 with respect to T1 (5338 ± 2860 dyne/s/cm^5^ at T1 vs 2198 dyne/s/cm^5^ at T2, *p* < 0.05) but in IR + F group, it remained higher at T2 than at BL (1745 ± 452 dyne/s/cm^5^ at BL vs 4223 ± 2636 dyne/s/cm^5^ at T2, *p* < 0.05). RVR in IR + F group at T2 was also higher than in the same time point of the C and C + F groups (4223 ± 2636 dyne/s/cm^5^ in IR + F vs 1645 ± 507 dyne/s/cm^5^ in C and 2198 ± 879 dyne/s/cm^5^ in C + F, *p* < 0.05). No significant changes were observed in the C and C + F groups.

### Renal oxygenation variables

**Fig. 2:**
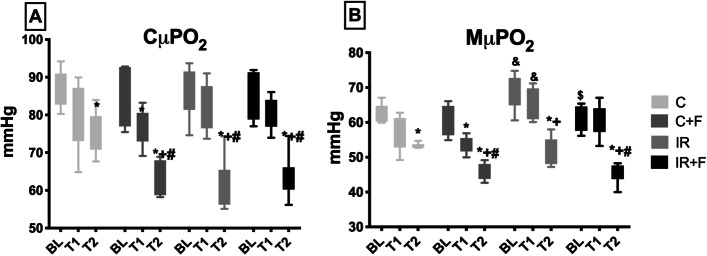
Alterations of the renal cortex (**A**) and medulla (**B**) partial oxygen pressure between the experimental groups and time points. Values are represented as mean ± SD, ^*^p < 0.05 vs. BL, ^+^*p* < 0.05 vs. T1, ^#^*p* < 0.05 vs. at same tome point of Control (C) group, ^&^ < 0.05 vs. at same time point of Control + Furosemide (C + F) group

CµPO_2_ and MµPO_2_ are shown in Fig. 2. IR caused a significant decrease in CµPO_2_ and MµPO_2_ at T2 compared to their BL values (CµPO_2_; 61.6 ± 6.8 vs 86 ± 6.6 mmHg and MµPO_2_; 51.1 ± 4.1 vs 68.7 ± 4.9 mmHg, *p* < 0.05). Furosemide administration did not alleviate this reduction in the IR + F group at T2 in compared to BL (45.6 ± 3 vs 60.2 ± 3.6 mmHg, *p* < 0.05). Additionally, furosemide without IR insult also decreased both CµPO_2_ and MµPO_2_ at T2 compared to the C group (CµPO2; 64.4 ± 4.6 vs 74.8 ± 5.6 mmHg and MµPO_2_; 46.1 ± 2.3 vs 53.3 ± 0.7 mmHg, *p* < 0.05).

**Fig. 3:**
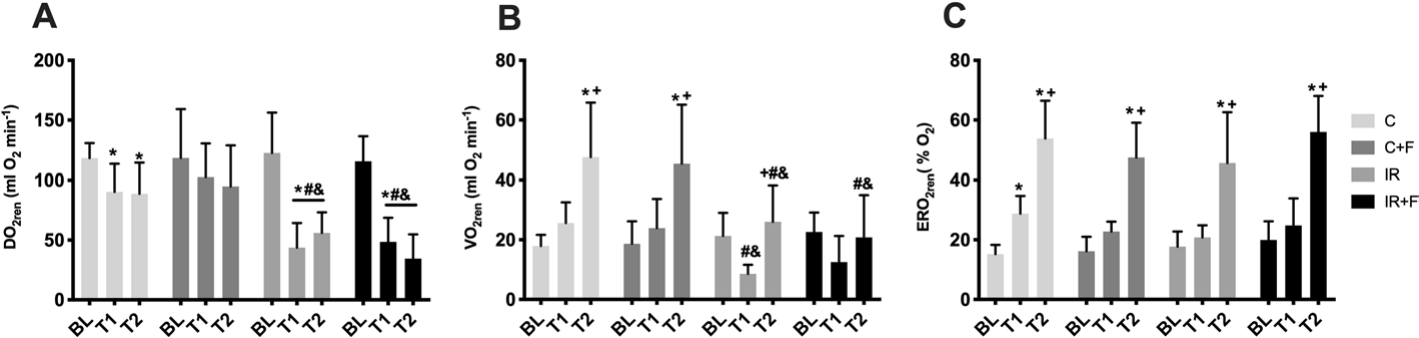
Renal oxygen deliver (DO_2ren_) (**A**), oxygen consumption (VO_2ren_) (**B**) and oxygen extraction ratio (ERO_2ren_) (**C**) between the experimental groups and time points. Values are represented as mean ± SD, ^*^p < 0.05 vs. BL, ^+^*p* < 0.05 vs. T1, ^#^*p* < 0.05 vs. at same tome point of Control (C) group, ^&^ < 0.05 vs. at same time point of Control + Furosemide (C + F) group

DO_2ren_, VO_2ren_ and ERO_2ren_ values are shown in Fig. 3. DO_2ren_ was significantly reduced in the IR and IR + F groups at T1 and T2 in comparison to their BL (IR; 43.5 ± 20.4 vs 122.3 ± 33.9 and IR + F; 48.4 ± 20.1 vs. 115.5 ± 20.9) (IR; 55.8 ± 17 vs 122.3 ± 33.9 and IR + F; 34.4 ± 20.3 vs 115.5 ± 20.9) (*p* < 0.05). It did not significantly change in the C + F group. Also, DO_2ren_ was significantly reduced in the IR and IR + F groups in compared with the C and C + F at both T1 (IR; 43.5 ± 20.4 and IR + F; 48.4 ± 20.1 vs. C; 90.3 ± 23.2 and C + F; 102.7 ± 27.7) and T2 (IR; 55.8 ± 17.1 and IR + F; 34.4 ± 20.3 vs C: 88.7 ± 25.7 and C + F: 94.8 ± 34 (*p* < 0.05) (Fig. 3A). VO_2ren_ was significantly increased in the C group at T2 compared to their BL (C; 47.5 ± 18.2 vs 17.9 ± 3.7 and C + F; 45.3 ± 19.7 vs 18.6 ± 7.5, *p* < 0.05). It did not significantly change in IR and IR + F groups at T2 in comparison to their BL. It was significantly lower in IR and IR + F groups at T2 in compared with the C and C + F (IR; 26 ± 12.2 and IR + F; 20.7 ± 14.1 vs C; 47.5 ± 18.2 and C + F; 45.3 ± 19.7) (*p* < 0.05) (Fig. 3B). There was not any statistically significant difference between the IR and IR + F at T2. ERO_2ren_ was significantly increased in all of the groups at T2 compared to their BL (C; 53.8 ± 12.6 vs 15.2 ± 3:

C + F; 47.4 ± 11.6 vs 16.1 ± 4.8: IR; 4.57 ± 17 vs 17.6 ± 5.1: IR + F; 56 ± 12 vs 19.9 ± 6.2) (*p* < 0.05). There was not any significant difference between groups at any timepoint (Fig. 3C).

### Renal function parameters and urine output

**Fig. 4:**
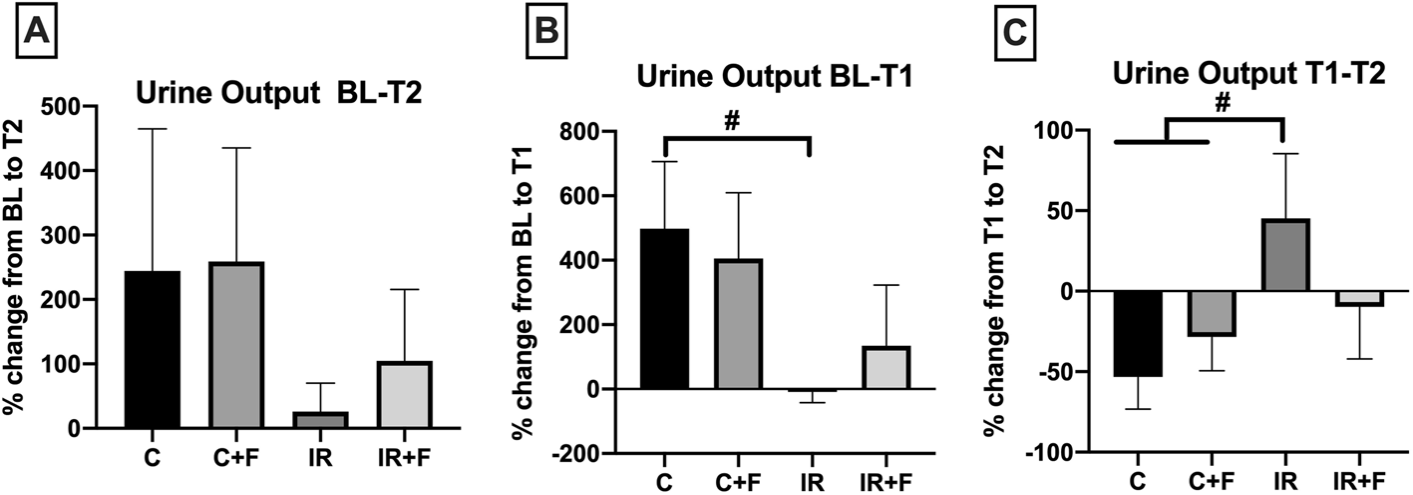
The percentage changes of urine output from BL-T2 (**A**), BL-T1 (**B**) and T1-T2 (**C**). Values represented as mean ± SD, ^#^*p* < 0.05 vs. control (C)

**Fig. 5:**
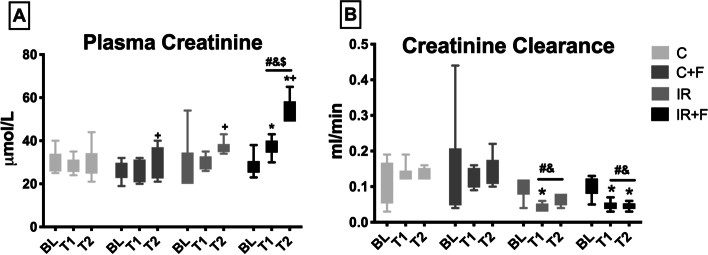
Alterations of plasma creatinine (**A**) and creatinine clearance (**B**) between the experimental groups and time points. Values are represented as mean ± SD, ^*^p < 0.05 vs. BL, ^+^*p* < 0.05 vs. T1, ^#^*p* < 0.05 vs. at same tome point of Control (C) group, ^&^p < 0.05 vs. at same time point of Control + Furosemide (C + F) group, ^$^*p* < 0.05 vs. at same time point of ischemia/reperfusion (IR) group

Renal function parameters are shown in Figs. 4, 5. There was no significant alteration of urine output percentage between the C (% 144 [86–440]), C + F (% 180 [106–468]), IR (% 23 [− 22 to 74]) and IR + F (% 47 [15–227]) groups from BL to T2 (Fig. 4A). IR caused a significant reduction in UO at T1 compared to the C group (− 8 ± 33 vs. 498 ± 207% *p* < 0.05) (Fig. 4B). While UO increased in IR group from T1 to T2 in comparison to the C (45 ± 40 vs. − 53 ± 40% *p* < 0.05), it was significantly reduced by furosemide administration after IR from T1 to T2 (+ 45 ± 40% vs. − 9 ± 32%, *p* < 0.05) (Fig. 4C).

Plasma creatinine was increased significantly at T2 compared to BL. The addition of furosemide did not improve creatinine clearance and, in contrast, it further increased plasma creatinine at T2, in the IR + F in compared to the IR only (54.83 ± 5.88 vs 37.17 ± 3.13 mmol/l, *p* < 0.05) (Fig. 5A). Creatinine clearance was significantly lower in the IR (0.04 ± 0.01 ml/min) and IR + F (0.05 ± 0.01 ml/min) groups in compared with the C (0.13 ± 0.02 ml/min) and C + F (0.12 ± 0.03 ml/min) groups at T1 (*p* < 0.05). This significance also persisted at T2 (0.06 ± 0.01 ml/min in the IR and 0.05 ± 0.01 ml/min in the IR + F vs 0.13 ± 0.01 ml/min in the C 0.13 ± 0.04 ml/min in C + F) (*p* < 0.05) (Fig. 5B).

**Fig. 6:**
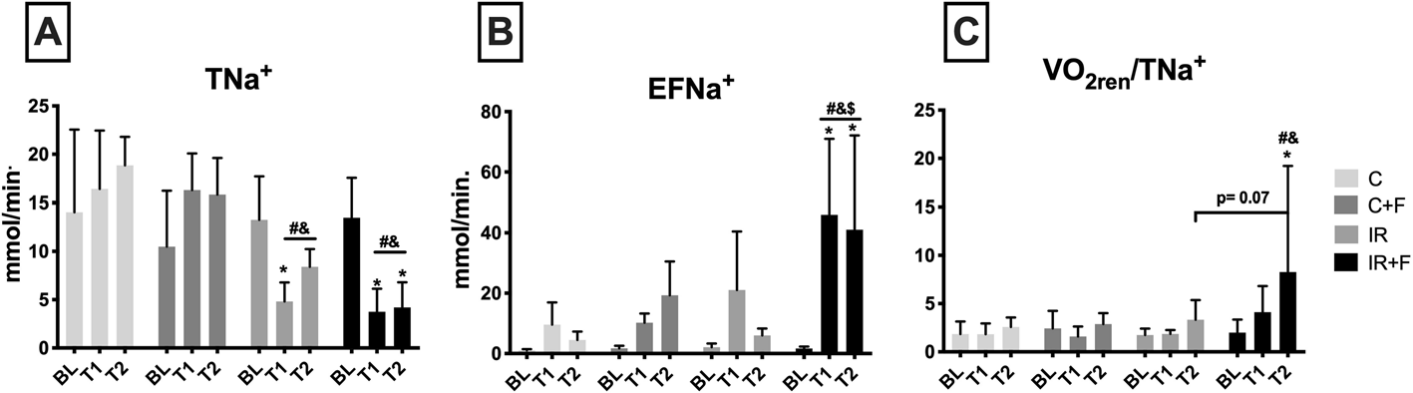
Renal sodium reabsorption (TNa^+^) (**A**), fractional sodium extraction (EFNa^+^) (**B**), and renal energy efficiency (VO_2ren_/TNa^+^) (**C**) between the experimental groups and the time points. Values are represented as Mean ± SD, ^*^p < 0.05 vs. BL, ^+^*p* < 0.05 vs. T1, ^#^*p* < 0.05 vs. at same tome point of Control (C) group, ^&^ < 0.05 vs. at same time point of Control + Furosemide (C + F) group, ^$^*p* < 0.05 vs. at same time point of ischemia/reperfusion (IR) group

Sodium handling is shown in Fig. 6. IR caused a significant reduction in TNa^+^. Furosemide administration did not affect TNa^+^ after IR. TNa^+^ was significantly reduced in the IR and IR + F at T2 in compared with the C and C + F groups (8.4 ± 1.8 and 4.2 ± 2.5 mmol/min vs 18.9 ± 3 and 15.8 ± 3.8 mmol/min, respectively) (*p* < 0.05) (Fig. 6A). IR alone did not cause a significant increase in EFNa^+^. However, EFNa^+^ was significantly increased in the IR + F group at T1 (46 ± 25 mmol/min) and T2 (41 ± 31 mmol/min) in comparison to the C (9.6 ± 7.4 and 4.5 ± 2.8 mmol/min), C + F (10.3 ± 3 and 19.3 ± 11.2 mmol/min) and the IR (21 ± 19.4 and 6 ± 2.3 mmol/min) groups (*p* < 0.05) (Fig. 6B). VO_2_/TNa^+^ ratio increased in the IR + F group at T2 compared to BL (1.9 ± 1.3 at BL vs 8.2 ± 10 at T2, *p* < 0.05), it was also higher in the IR + F group than in the C and C + F groups (2.5 ± 0.9 in the C and 2.8 ± 1.3 in C + F vs 8.2 ± 10 in IR + F group, *p* = 0.07), but this rise did not reach statistical significance in comparison to the IR group (3.3 ± 2 in IR vs 8.2 ± 10 in IR + F, *p* = 0.07) (Fig. 6C).

### Biochemical results

**Fig. 7:**
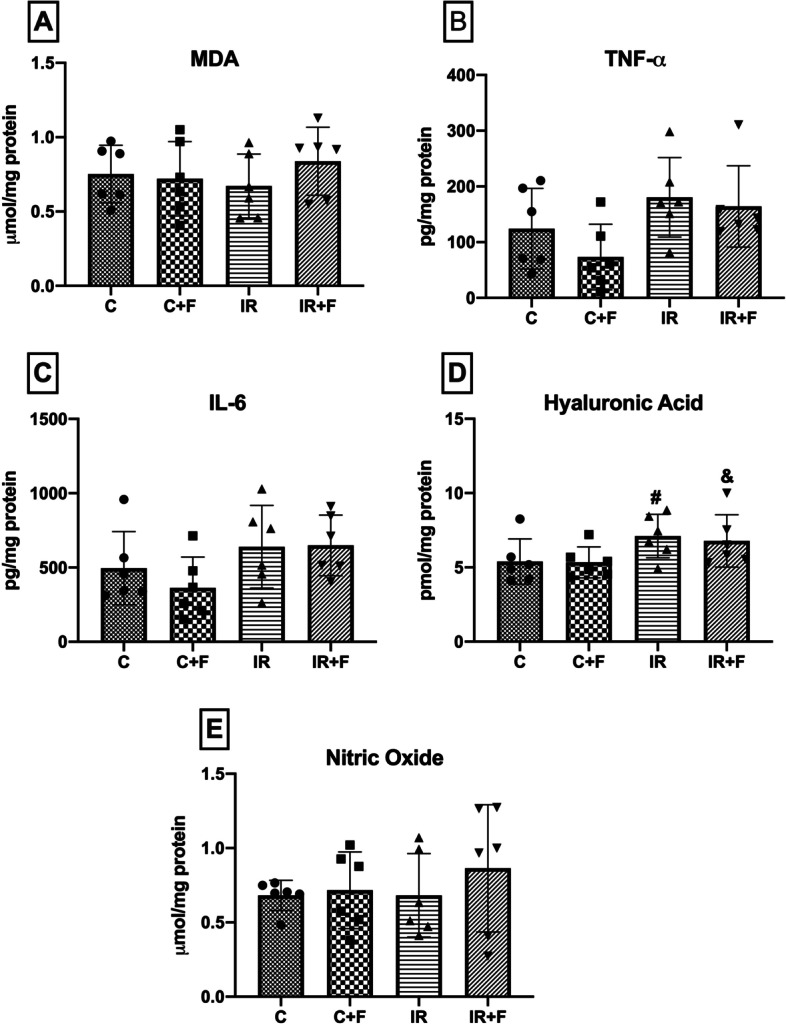
Malondialdehyde (MDA) (**A**), tumor necrosis factor-alpha (TNF-α) (**B**), interleukine-6 (IL-6) (**C**), hyaluronic acid (**D**), and nitric oxide (**E**) levels between the experimental groups. Values are represented as mean ± SD, ^#^*p* < 0.05 vs. Control (C), ^&^*p* < 0.05 vs. Control + Furosemide (C + F) group

There was not any change in plasma anion gap, pH, PaO_2,_ and the Hct between groups (Table 1). MDA, TNF-α, IL-6, and NO levels showed a non-significant alteration after IR compared with the (Fig. 7A–C, E). Hyaluronic acid levels increased in IR (*p* < 0.05) group compared with the C (*p* < 0.05). Hyaluronic acid also increased significantly in IR + F group compared with the C + F group (*p* < 0.05) (Fig. 7D).

**Table 1:** Blood oxygen, electrolytes, and biochemistry

	BL	T1	T2
*PaO*_*2*_* (mmHg)*			
Control	196.33 ± 12.31	200 ± 14.48	177.17 ± 12.02
C + F	208.17 ± 5.78	208.67 ± 5.85	198.33 ± 7.37
IR	197.83 ± 20.01	195.5 ± 12.05	179.83 ± 7.83
IR + F	188.67 ± 13.62	187.17 ± 9.62	184.50 ± 5.36
*Hct (% RBC)*			
Control	50.33 ± 1.86	48.33 ± 2.88	40.83 ± 3.92^*+^
C + F	47.5 ± 1.05^#^	46.83 ± 1.94	40.17 ± 1.47^*+^
IR	50.17 ± 3.06	47 ± 2.97^*^	41.33 ± 2.8^*+^
IR + F	49 ± 1.79	47.17 ± 5.12	41.5 ± 4.85^*+^
*Anion gap(mmol/L)*			
Control	18.85 ± 0.56	20.88 ± 1.54	20.48 ± 3.45
C + F	18.10 ± 1.45	19.85 ± 1.27	17.15 ± 1.64
IR	19.93 ± 1.67	21.82 ± 2.14	21.08 ± 1.09
IR + F	18.77 ± 1.26	22.17 ± 1.28	20.90 ± 2.17
*pH*			
Control	7.41 ± 0.04	7.40 ± 0.02	7.42 ± 0.03
C + F	7.42 ± 0.03	7.40 ± 0.03	7.45 ± 0.03
IR	7.42 ± 0.03	7.37 ± 0.03	7.42 ± 0.02
IR + F	7.43 ± 0.02	7.41 ± 0.10	7.39 ± 0.03

Values are represented as mean ± SD. *p < 0.05 vs. BL, ^+^ *p* < 0.05 vs. T1, ^#^*p* < 0.05 vs. C, ^&^*p* < 0.05 vs C + F

### Renal histopathology

**Fig. 8:**
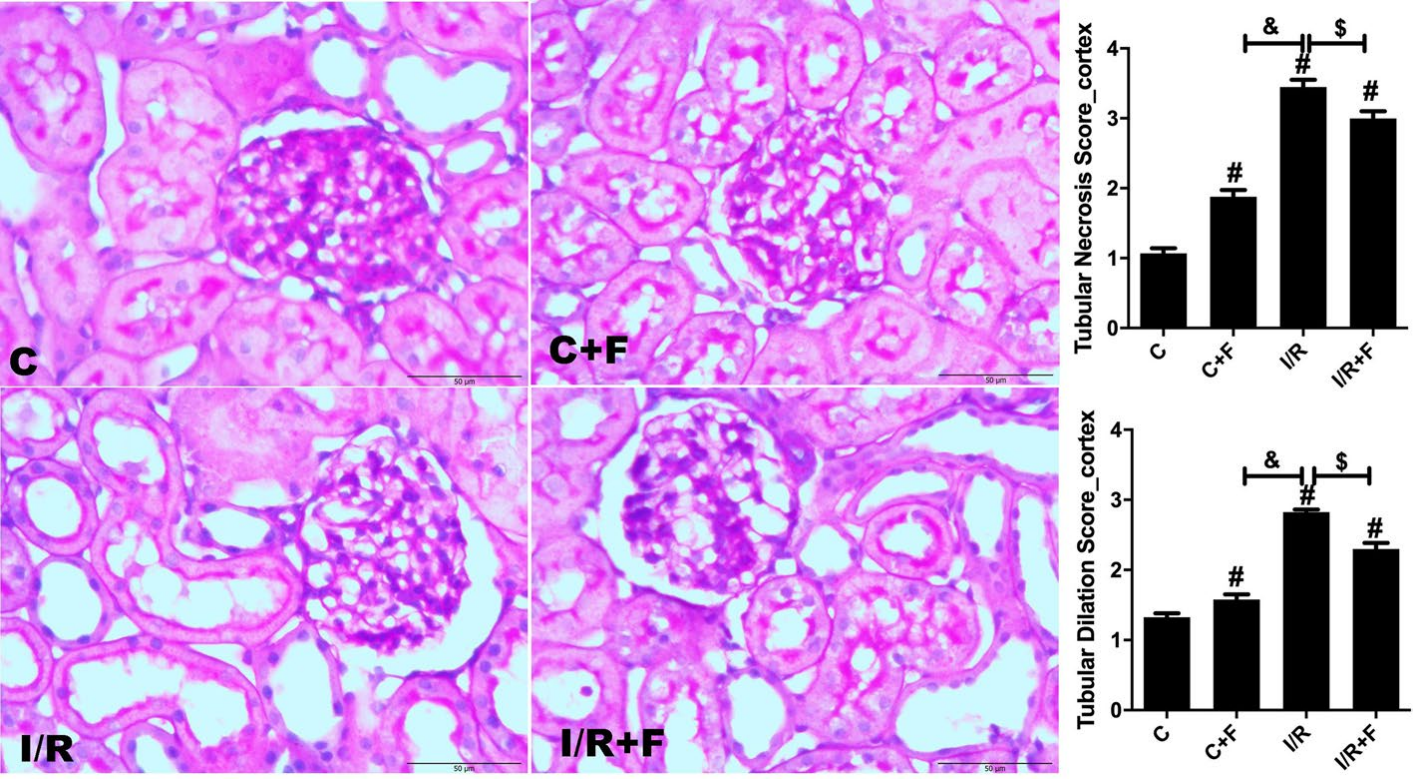
Renal cortical tubular dilation and necrosis scores with representative figures. PAS– hematoxylin images: C; control, C+F; control + furosemide, I/R; ischemia/reperfusion, I/R+F; ischemia/reperfusion + furosemide. Values represented as mean ± SD, ^#^*p* < 0.05 vs. control (C)), ^&^*p* < 0.05 vs. control + furosemide (C + F), ^$^*p* < 0.05 vs. ischemia/reperfusion (IR)

**Fig. 9:**
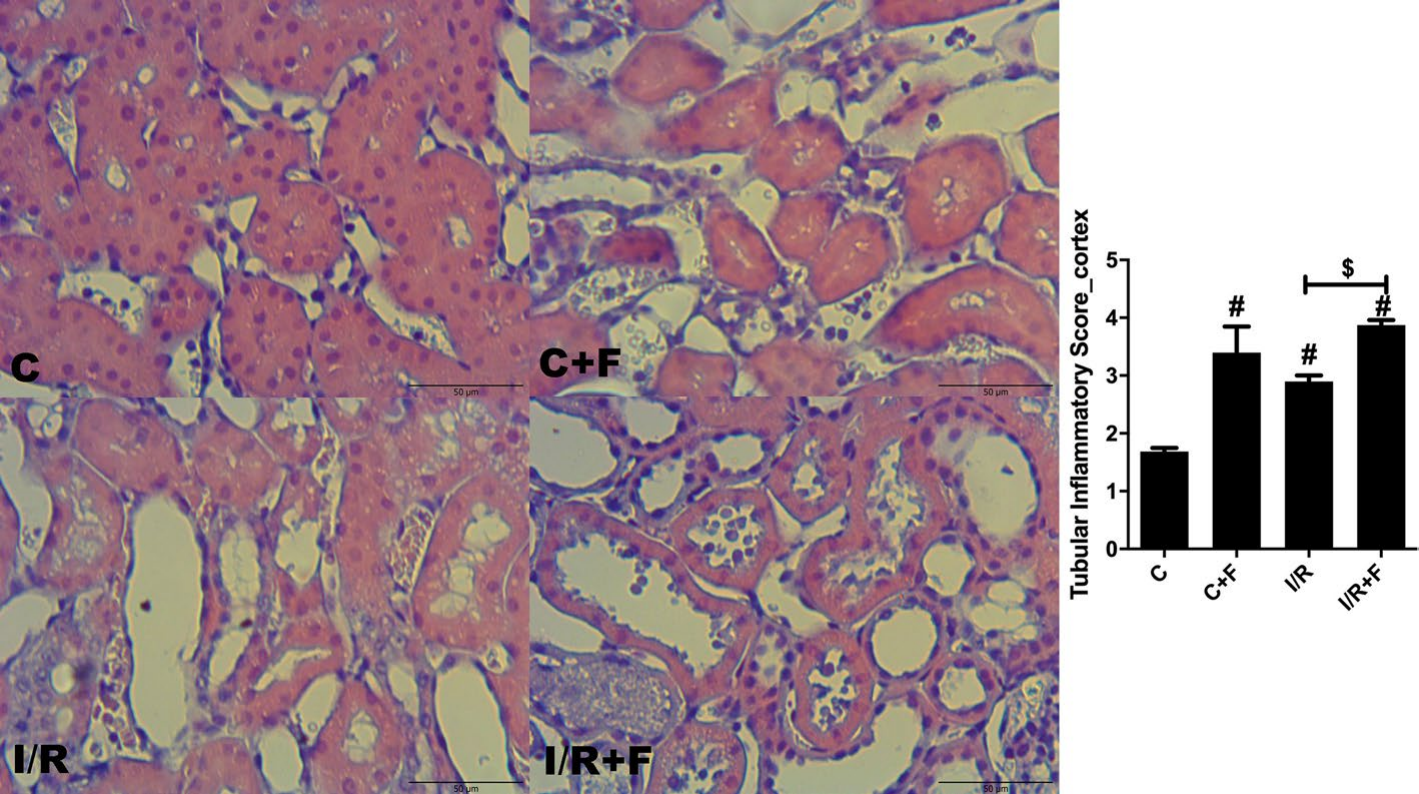
Renal cortical inflammatory scores and representative figures. Hematoxylin–eosin images: C; control, C+F; control + furosemide, I/R; ischemia/reperfusion, I/R+F; ischemia/reperfusion + furosemide. Values represented as mean ± SD, ^#^*p* < 0.05 vs. control (C), ^&^*p* < 0.05 vs. control + furosemide (C + F), ^$^*p* < 0.05 vs. ischemia/reperfusion (IR)

**Fig. 10:**
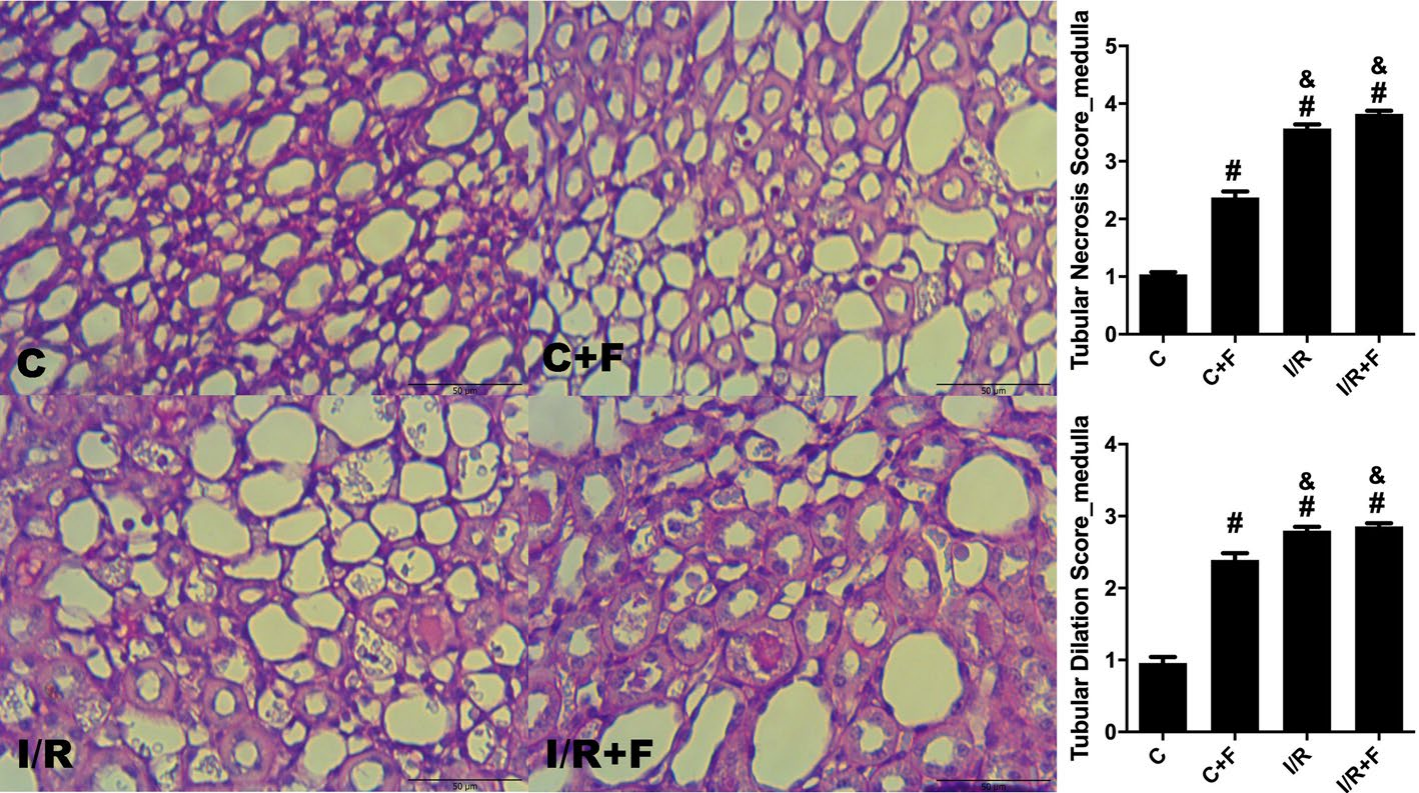
Renal medullary tubular dilation and necrosis scores with representative figures. PAS–hematoxylin images: C; control, C+F; control + furosemide, I/R; ischermia/reperfusion, I/R+F; ischemia/reperfusion + furosemide. Values represented as mean ± SD, ^#^*p* < 0.05 vs. control (C), ^&^*p* < 0.05 vs. control + furosemide (C + F)

**Fig. 11:**
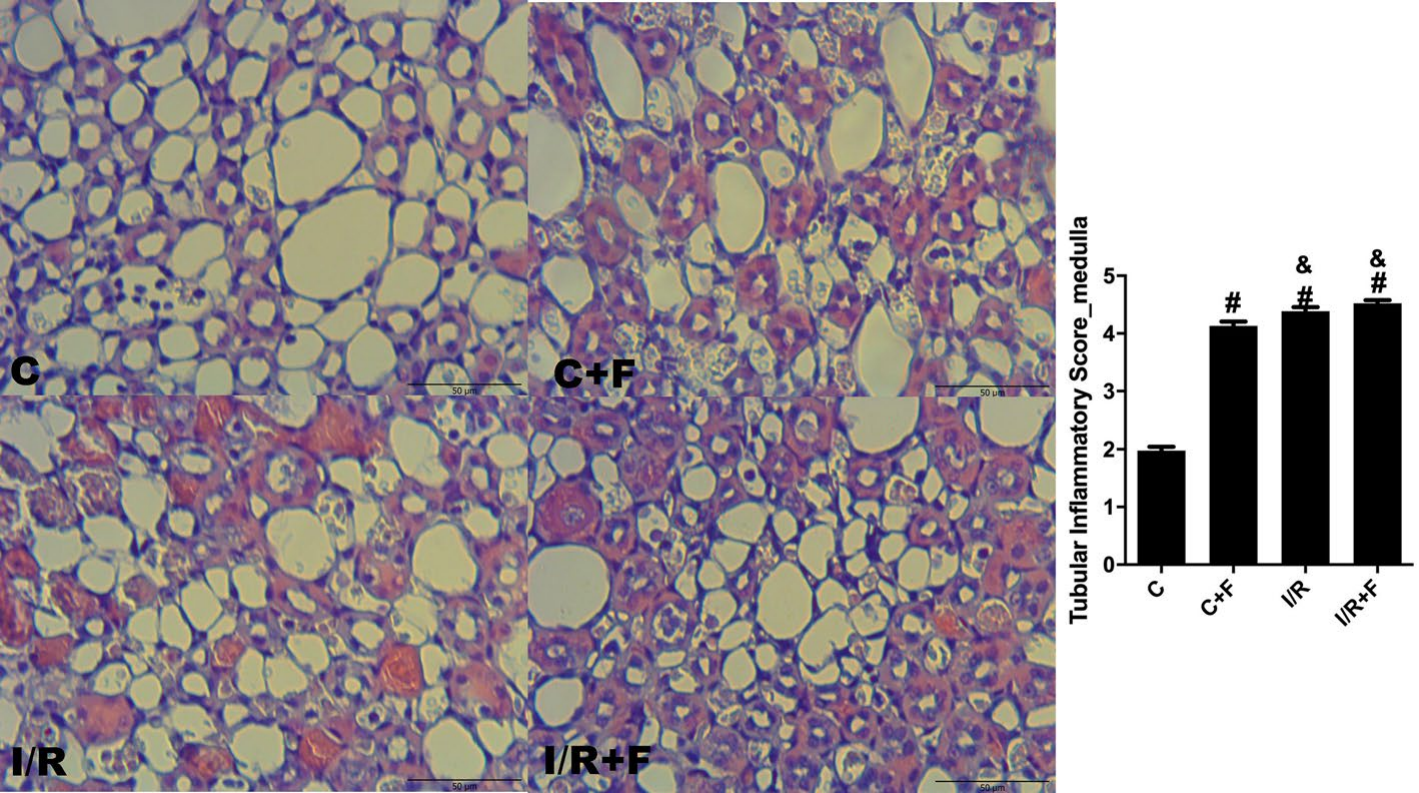
Renal medullary inflammatory scores and representative figures. Hematoxylin–eosin images: C control, C+F; control + furosemide, I/R; ischemia/reperfusion, I/R+F; ischemia/reperfusion + furosemide. Values represented as mean ± SD, ^#^*p* < 0.05 vs. control (C), ^&^*p* < 0.05 vs. control + furosemide (C + F)

Renal cortical dilatation, necrosis and inflammatory cell migration were increased in the C + F, IR and IR + F groups in comparison to the C (*p* < 0.05) (Figs. 8, 9). While furosemide led to decrease the tubular dilatation and necrosis in the cortex of IR + F group (Fig. 8), increased the inflammatory cell migration with respect to the IR group (*p* < 0.05) (Fig. 9). In medulla, tubular necrosis, dilation and inflammatory score were evident in the IR, C + F and IR + F groups in comparison with the C group –(*p* < 0.05) (Figs. 10, 11).

## Discussion

The previous studies evaluating the use of furosemide after kidney injury observed inconsistent results. It was hypothesized that furosemide administration following IR-induced AKI may have a beneficial role in renal tissue oxygenation, function and inflammation by means of facilitating oxygenation or reducing oxygen consumption. Our important finding is that furosemide did not improve either perfusion or oxygenation after IR-induced AKI in rats. Instead, furosemide aggravated the RVR and plasma lactate level. To complicate things further, furosemide exacerbated the oxygen utilization efficiency following IR insult. Moreover, furosemide administration augmented the renal injury and reduced the renal oxygenation even in the control condition.

Furosemide is commonly used to promote diuresis. It is also employed to prevent or limit kidney injury by uncoupling sodium absorption, thus conserving energy and oxygenation [19]. To date, however, there has been little definitive evidence that it is so [9, 20–27]. Additionally, diuretic use was independently associated with an increased risk of AKI among the hospitalized Covid-19 patients which is a disease characterized with widespread inflammation [11, 28, 29]. Besides, we demonstrated that furosemide disrupts kidney perfusion after IR insult without effecting the systemic hemodynamic parameters. Despite RBF was decreased in IR groups, much of the reduction in the perfusion was driven by an increased RVR following furosemide administration. As a result, we showed that furosemide has vasoconstrictive effects on the renal vascular bed after ischemia. Indeed, Castrop et al., demonstrated that furosemide increases renin secretion which subsequently activates renin–angiotensin–aldosterone system and causes afferent arteriole vasoconstriction [30]. Despite several studies indicating otherwise, such as improved medullary oxygenation without any increase in the medullary blood flow [31], we observed a reduction of oxygen availability in cortex and medulla after IR but only the medullary oxygenation was marginally decreased in IR groups received furosemide in compared to the Control group. An increased creatinine levels in IR group following furosemide administration injury indicates an aggravated renal dysfunction. Additionally, we also showed that furosemide has some negative effects of cortical and medullary oxygenation in the control condition without effecting the RVR. This may be explained by an increased renal oxygen shunting [6] in both IR and Control groups received furosemide. These results may also confirm that furosemide has an effect not only in the renal medullary perfusion and oxygenation, but also in the cortex.

It is assumed that nephron workload is the primary determinant of oxygen distribution in the kidney [32]. Renal medulla is the most susceptible part hypoxia due to its limited blood supply and high energy consumption in comparison to the cortex [33]. Therefore, reduced sodium delivery to distal parts of the nephron due to severely decreased glomerular filtration as a result of disrupted renal blood flow should simultaneously reduce the oxygen requirement of medullary tubules and increase its availability elsewhere [32]. Indeed, Ow et al. showed that renal tissue oxygenation is maintained by a reduction in oxygen consumption even 5 days after severe IR insult and cellular hypoxia was negligible in rats that did not receive furosemide [34]. However, perhaps the most striking finding in our study was reduced medullary oxygenation after furosemide and IR, which was shown previously in furosemide naïve rats [17, 34–36]. Inhibition of the electrolyte absorption in the TAL by furosemide exposes distal parts of the nephron to increased workload to compensate the electrolyte loss and ensure homeostasis [7]. This compensation is overwhelmed in the injured kidney as shown by disproportionately increased Na^+^ excretion compared to absorption despite reduced oxygen consumption in compared to the rats that did not receive furosemide after the IR insult. Additionally, VO_2_/TNa^+^ is increased following furosemide administration indicating impaired oxygen utilization efficiency per Na^+^, as also reported in the previous studies [37–41]. Altogether, these results indicate that furosemide may disrupt the adaptive responses against hypoxia in the injured kidney.

It is well known that IR generates reactive oxidant and nitrosative species forming peroxynitrite [42–44]. Peroxynitrite interaction triggers propagation of inflammation, loss of the local autoregulation of blood flow and subsequent tissue injury [33, 42–44]. Silbert and co-workers founded that use of furosemide caused an increase in oxidative stress depending on the severity of AKI [12]. In this study, we showed a distinct cortical and medullary injury in term of tubular dilatation, necrosis and inflammation caused by IR. In parallel to our findings, Chu et al., demonstrated that after 45 min renal arterial clamping resulted in severe tubular dilatation, necrosis, and high kidney injury molecule-1 (KIM-1) expression [18]. Moreover, they also showed that the use of empagliflozin as a sodium glucose cotransporter-2 (SGLT-2) inhibitor, which inhibits the SGLT-2 in proximal tubules to reduce Na^+^ and glucose re-uptake, in IR-induced AKI restored the renal tissue damage and KIM-1 expression [18]. In compared with SGLT-2 inhibitors, furosemide plays a role in TAL in Henle which has a higher oxygen requirement than the proximal tubules due to its function on great deal of salt and water reabsorption, and urine condensation. Thus, it seems that the inhibition of Na^+^ K^+^ and Cl^−^ transport, and water reabsorption in TAL by furosemide results no beneficial effects on medullar damage as shown in this study. In fact, Rokuten et al., showed that use of the furosemide after myocardial infarction on rats were strongly associated with renal damage and mortality in compared to the placebo and the experimental group received angiotensin converting enzyme (ACE) inhibitor [45]. In present study, despite furosemide increased the inflammatory cell migration in renal cortex, it reduced the renal tubular dilatation and necrosis in comparison to the IR alone. However, furosemide showed no beneficial effects on the renal medullar dilation and necrosis after IR insult. This discrepancy in the effects of furosemide on cortex and medulla can be explained by the structural and functional differences of renal medulla which includes abundant of active furosemide receptor, limited blood supply, higher workload and oxygen consumption with respect to cortex. Importantly, furosemide also caused a distinct cortical and medullary damage in term of tubular dilation, cell necrosis and inflammatory cell migration in the control conditions. Therefore, this result indicates that furosemide can also injure the kidney without any pre-existing damage. These results may indicate that overwhelming effects of furosemide on medulla may be also overlapped by IR injury on its own because of the severity of AKI model we used in this study. However, surrogates of tissue inflammation and oxidative stress markers were not altered in our study. Plasma hyaluronic acid, a glycocalyx degradation biomarker, was significantly increased after IR and did not decrease after furosemide administration. Together with the renal oxygenation, plasma creatinine level, renal vascular resistance and sodium handling results, aggravation of hypoxia-induced tissue damage is the main factor causing extra renal injury following furosemide administration.

## Conclusion

In conclusion, the present study was designed to determine the effect of furosemide on the kidneys after IR insult. Contrary to the expectations, our findings indicate that furosemide does not improve either the cortical and medullary microvascular oxygenation or renal function. Furthermore, it may even aggravate AKI by disrupting Na^+^ reabsorption, oxygen utilization efficiency, tissue perfusion and increasing hypoxic injury in the kidney. Taken together, our findings contribute in several ways to our understanding of AKI and provides a basis for future clinical studies which aims to prevent or limit the extent of renal injury following a hypoxic insult.

Despite its exploratory nature, our study offers insight into the relationship between furosemide and AKI. It is possible that these results may not fully apply to AKI in humans encountered after scenarios mentioned in the introduction. Therefore, there would be a certain need for clinical trials to better understand the possible link between hypoxia and oxygen utilization efficiency.

## Data Availability

Not applicable.

## Abbreviations

AKI: Acute kidney injury
F: Furosemide
IR: Ischemia/reperfusion
MAP: Mean arterial pressure
RBF: Renal blood flow
RVR: Vascular resistance
DO_2ren_: Renal oxygen delivery
VO_2ren_: Renal oxygen consumption
TNa^+^: Sodium reabsorption
VO_2_/TNa^+^: Oxygen utilization efficiency
EF_Na_: Excretion fraction of Na^+^
O_2_ER_ren_ (%): Renal oxygen extraction ratio
CμO_2_: Cortical microvascular oxygen pressures,
MμO_2_: Medullary microvascular oxygen pressures
UO: Urine output
Ccr: Creatinine clearance
TAL: Tick ascending limp
PO_2_: Partial oxygen pressure
FiO_2_: Inspired oxygen pressure
PaO_2_: Arterial oxygen pressure
PrvO_2_: Renal venous oxygen pressure
SaO_2_: Arterial oxygen saturation
SrvO_2_: Renal venous oxygen saturation
AOC: Arterial oxygen content
RVOC: Renal venous oxygen content
mmHg: Millimeter mercury
kPa: Kilo pascal
TNF-α: Tumor necrosis factor- alpha
IL-6: Interleukin-6
HA: Hyaluronic acid
MDA: Malondialdehyde
NO: Nitric oxide
